# Three options for citation tracking: Google Scholar, Scopus and Web of Science

**DOI:** 10.1186/1742-5581-3-7

**Published:** 2006-06-29

**Authors:** Nisa Bakkalbasi, Kathleen Bauer, Janis Glover, Lei Wang

**Affiliations:** 1 Yale University Library, 130 Wall St., P.O. Box 208240, New Haven, CT 06520-8240, USA; 2 Cushing/Whitney Medical Library, Yale School of Medicine, 333 Cedar St. P.O. Box 20804, New Haven, CT 06520-8014, USA

## Abstract

**Background:**

Researchers turn to citation tracking to find the most influential articles for a particular topic and to see how often their own published papers are cited. For years researchers looking for this type of information had only one resource to consult: the Web of Science from Thomson Scientific. In 2004 two competitors emerged – Scopus from Elsevier and Google Scholar from Google. The research reported here uses citation analysis in an observational study examining these three databases; comparing citation counts for articles from two disciplines (oncology and condensed matter physics) and two years (1993 and 2003) to test the hypothesis that the different scholarly publication coverage provided by the three search tools will lead to different citation counts from each.

**Methods:**

Eleven journal titles with varying impact factors were selected from each discipline (oncology and condensed matter physics) using the Journal Citation Reports (JCR). All articles published in the selected titles were retrieved for the years 1993 and 2003, and a stratified random sample of articles was chosen, resulting in four sets of articles. During the week of November 7–12, 2005, the citation counts for each research article were extracted from the three sources. The actual citing references for a subset of the articles published in 2003 were also gathered from each of the three sources.

**Results:**

For oncology 1993 Web of Science returned the highest average number of citations, 45.3. Scopus returned the highest average number of citations (8.9) for oncology 2003. Web of Science returned the highest number of citations for condensed matter physics 1993 and 2003 (22.5 and 3.9 respectively). The data showed a significant difference in the mean citation rates between all pairs of resources except between Google Scholar and Scopus for condensed matter physics 2003. For articles published in 2003 Google Scholar returned the largest amount of unique citing material for oncology and Web of Science returned the most for condensed matter physics.

**Conclusion:**

This study did not identify any one of these three resources as the answer to all citation tracking needs. Scopus showed strength in providing citing literature for current (2003) oncology articles, while Web of Science produced more citing material for 2003 and 1993 condensed matter physics, and 1993 oncology articles. All three tools returned some unique material. Our data indicate that the question of which tool provides the most complete set of citing literature may depend on the subject and publication year of a given article.

## Background

Many researchers have an interest in finding citation information about a given article – both how many times the article is cited and who is citing that article. This may be for the completeness of a literature search, or perhaps to find how often his or her own publications are cited. Eugene Garfield made possible the widespread use of citation analysis in academe through his creation of three citation indices: Science, Humanities and Social Science Citation Indices, which were combined and transformed into an electronic version called the Web of Science. These indices were based on the concept that a carefully selected subset of journals would produce the majority of important citing literature for any given article. Citation analysis has real world implications: for good or bad, citedness is considered in grants, hiring and tenure decisions. For many reasons professors and researchers may want to demonstrate the impact of their work and citation analysis is one way (albeit a controversial one [1-3]) to accomplish this. For many years Web of Science had a virtual monopoly on the provision of citedness tracking. Late in 2004 two competitors to Web of Science emerged – Google Scholar and Scopus.

The Internet search giant Google sponsored the creation of Google Scholar, a tool that attempts to give users a simple way to broadly search the scholarly literature. Google Scholar uses a matching algorithm to look for keyword search terms in the title, abstract or full text of an article from multiple publishers and web sites (Google Scholar does not share the specifics of how this algorithm works). The number of times a journal article, book chapter, or web site is cited also plays an important part in Google Scholar's ranking algorithm. Search results are displayed so that the more cited and highly relevant articles rise to the top of the set. This varies from the more traditional default "reverse chronological" order employed by most scholarly databases. Google Scholar neither lists the journal titles it includes, nor the dates of coverage; although they have indicated that they have agreements with most major publishers (except Elsevier). Another area of difference for Google Scholar is that unlike most scholarly research databases, it looks beyond journal literature to cover other modes of scholarly communication. Other sources covered in Google Scholar include preprint servers such as arXiv (physics) and government and academic Web sites. Google Scholar does not state how a Web site qualifies for inclusion in its searches.

At approximately the same time that Google Scholar was made public, Elsevier introduced Scopus, an indexing and abstracting service that contains its own citation-tracking tool. Scopus indexes a larger number of journals than Web of Science, and includes more international and open access journals. Citation coverage however only dates to 1996 (abstracts, but not citation coverage, are available back to 1966 for some journals.) Scopus includes its own Web search engine, Scirus. Scirus results are presented separately from other Scopus journal results. Also, material from Scirus does not figure into citation counts for Scopus journal records. Table 1 provides a comparison summary of features in Web of Science, Scopus, and Google Scholar.

**Table 1:** Comparison of features in Web of Science, Scopus, and Google Scholar

	**Web of Science**	**Scopus**	**Google Scholar**
Indexing and abstracting	Yes	Yes	No
Years covered-journals	1900 to present (Science) 1956-present (Social Science) 1975-present (Arts and Humanities)	1966 to present for some journals, but many date back to 1996 to present	Not revealed
Years covered-citations	1900 to present	1996 to present	Not revealed
Fee-based	Yes	Yes	No
Contents	9300 journals (Science, Social Science and Arts and Humanities)	15,000 journals (Science and Social Science)	Not revealed

Citation analysis has been the focus of research and discussion for decades. Much has been written about citation analysis techniques [18-29], application to different disciplines [1,28,30], and controversies surrounding the use of citation analysis and journal impact factors to gauge the value and impact of a given journal title or the corpus of a given author [1-3]. With the introduction of Scopus and Google Scholar, there have been many recent articles that include careful analysis of the features of each individual tool as well as comparisons among two or more of these tools, and others (for example, PubMed and Scirus) [9-17]. While these articles discuss the general characteristics and report the results of sample searches the authors have completed, they do not systematically review the citation analysis functions. In a 2005 study analyzing Google Scholar, Noruzi [14] briefly compared citation counts for two products – Google Scholar and Web of Science – in the field of webometrics. First, the author selected the first article to establish the word "webometrics" [18], and provided the "times cited" for both the Web of Science and Google Scholar. The author then compared the number of unique and overlapping citations to this one article in each product. Noruzi also looked at the citation counts for the "most-cited" articles in the field by conducting a search on the term "webometrics or webometric" in each product.

There are inherent problems using subject searches as a comparison measure because of the differences in how Web of Science, Google Scholar and Scopus perform searches. For example, Web of Science does not automatically search for common word variations, while Scopus and Google Scholar do. Similar keyword searches in Scopus and Web of Science often return relatively small result sets (less than one hundred records), while the same search in Google Scholar may return hundreds of results. For example, a search for the phrase "complementary medicine" with the word "obesity" returns 9 results in Scopus, 6 in Web of Science and 596 results in Google Scholar.

Citation tracking of known articles as a comparison method avoids the inconsistencies in subject searching. In a preliminary study Bauer and Bakkalbasi [31] examined the citation counts for these three tools for articles from the Journal of the American Society for Information Science and Technology (JASIST) published in 1985 and 2000. They found that older material appears best covered by the Web of Science, although this was not confirmed statistically due to the small size of the dataset. For the newer material citation counts were higher in Google Scholar than either Web of Science or Scopus, while there was no statistical difference between the citation counts reported by Web of Science and Scopus. The authors recommended a larger, more robust study.

In attempting to provide a more robust study, this paper looks at a known set of articles, and examines the number of citing articles and other material returned by each of the three search tools for that discrete set, thus removing the ambiguity inherent in subject searches. In this way the study produces data sufficient to test the hypothesis that the different scholarly publication coverage provided by the three search tools will lead to different citation counts from each. In selecting a set of articles to work with, we decided that we should also account for the variations in the publication habits for various disciplines [4-8]. Thus we chose two disciplines to investigate that we suspected were following different publication patterns. One, physics, has largely embraced the use of preprint servers for the early dissemination of research literature, while a second discipline, medicine, has not. The subjects were narrowed to condensed matter physics (henceforth referred to as CM physics) and oncology. Sets of known articles from each discipline were selected from both 1993 (before e-publishing dominated scientific disciplines) and 2003 (well into the e-publishing era).

This approach of working with sets of known articles and looking for citing material mirrors the experience of the searcher who is interested in finding citing references to a known article. What can this researcher expect from this new landscape that includes the familiar indices from the Web of Science with the new territory of Scopus and Google Scholar?

## Methodology

### Sample

The sampling process included two steps: first the selection of journal titles from each discipline (oncology and CM physics) and second the selection of articles from those journals. In the first step, we retrieved 123 journals listed in the "Oncology" category and 60 journals listed in the "Physics, Condensed Matter" category, using the 2004 Journal Citation Reports (JCR) database. Eleven journals from each category were selected using systematic sampling technique to ensure the sample contained an even distribution of journals across all levels of impact factors. To draw the sample all titles were ranked from highest to lowest by impact factors. Then title selection began with the first title and was expanded to include every n^th ^subsequent title where n, the sampling interval, was calculated as:

n = Population size/Sample size

Tables 2 and 3 furnish a list of the titles selected for the study.

**Table 2:** Oncology titles

**Journal Title**	**Impact Factor**
CA – A Cancer Journal for Clinicians	44.515
Journal of the National Cancer Institute	13.856
Advances in Cancer Research	6.200
Neoplasia	4.377
Cancer Immunology, Immunotherapy	3.52
Breast Cancer Research	2.975
BMC Cancer	2.290
Cancer Investigation	1.935
American Journal of Clinical Oncology	1.703
Chemotherapy	1.248
International Journal of Biological Markers	0.929

**Table 3:** CM physics titles

**Journal Title**	**Impact Factor**
Surface Science Reports	21.350
Journal Of The Mechanics And Physics Of Solids	3.443
Physical Review B	3.075
Semiconductor Science And Technology	2.152
Interface Science	1.639
(Incorporated Into Journal Of Materials Science As Of 2004)	
European Physical Journal B	1.426
Journal Of Physics And Chemistry Of Solids	0.988
Physics Of The Solid State	0.724
(An English Translation Of The Journal Fizika Tverdogo Tela)	
Phase Transitions	0.581
Solid State Technology	0.431
International Journal Of Modern Physics B	0.361

Articles from years 1993 and 2003 were selected as the population from which to draw the sample. The second step of the sampling process began by retrieving all the articles published in the selected eleven titles for both years using Web of Science, INSPEC, and PubMed. All editorial materials, notes, biographical items, corrections, letters, book reviews, and news items were removed from the dataset before the sampling, as these items were not of primary concern to the study. Using stratified random sampling to allow a proportional representation of each journal, a random sample of articles from each journal was drawn according to the ratio of articles in a given journal to the total number of articles. This resulted in four sets of varying sizes: 234 and 259 for oncology 1993 and 2003, respectively; and 358 and 364 for CM physics 1993 and 2003 respectively. The set for CM physics was larger mainly because of the inclusion in the sample of Physics Review B, which publishes thousands of articles each year. These four sets of articles would be used to gather citation counts from Web of Science, Scopus, and Google Scholar.

To create sets for an examination of the citing references for articles published in 2003, fifty articles from the sets for oncology and CM physics 2003 were tagged for inclusion in two subsets. Between three and five articles from each journal title were included in these subsets.

### Data collection

To construct the dataset author names, article title, source, volume number, and issue number were entered in a spreadsheet. Then during the week of November 7–12, 2005, citation counts were extracted for each research article from three sources: Web of Science, Scopus, and Google Scholar. The absence of some articles from any one of the three databases resulted in the elimination of 16 (7%) records from oncology 1993, 6 (2%) records from oncology 2003, and 18 (5%) records from CM physics 2003. Missing data from Scopus for CM physics 1993 resulted in a dataset too small to use for statistical significance. Thus Scopus was excluded from further analysis for this particular subject and year.

To augment the information gathered for citation counts, the actual citing references for the fifty articles in each of the two subsets of articles from 2003 oncology and CM physics were gathered from Web of Science, Scopus, and Google Scholar, resulting in two sets of citing references totaling 296 for CM physics and 614 for oncology.

## Results

Table 4 displays the descriptive statistics of the citation counts from each of the three resources. For oncology 1993, Web of Science returned the highest average number of citations, 45.3. Scopus returned the highest average number of citations (8.9) for oncology 2003. Web of Science returned the highest number of citations for CM physics 1993 and 2003 (22.5 and 3.9, respectively).

**Table 4:** Descriptive statistics for citation counts

		**Oncology**			**CM physics**		
		Google Scholar	Scopus	Web of Science	Google Scholar	Scopus	Web of Science
		
1993	Mean	20.8	35.4	45.3	10.3	N/A	22.5
	St. Dev.	37.9	60.7	77.4	20.7	N/A	32.5
							
2003	Mean	6.2	8.9	8.3	2.2	2.2	3.9
	St. Dev.	8.0	12.0	10.9	3.7	2.7	4.9

The hypothesis of the study is that the different scholarly publication coverage provided by the three search tools will lead to different citation counts from each. In addition, scholarly publication varies, encompassing many document dissemination methods depending on the subject discipline, and these differences will further be reflected in different citation counts for the three tools. We began by examining the following hypothesis:

H_o_: There is no difference among the citation counts extracted from the three resources.

H_a_: A difference exists among the citation counts extracted from the three resources.

Since the citation counts were highly skewed and the underlying assumptions for a parametric test were not met, a Friedman test, the non-parametric equivalent of the repeated measure ANOVA, was run for each discipline/year. Table 5 displays the summary results. The data showed a significant difference in the mean citation rates for at least one database.

**Table 5:** Friedman test results

		**Oncology 1993 (n = 218)**	**Oncology 2003 (n = 253)**	**CM Physics 2003 (n = 346)**
Mean Ranks				
	Google Scholar	1.18	1.51	1.68
	Scopus	2.07	2.38	1.86
	Web of Science	2.75	2.11	2.47
Test Statistics				
	Chi-Square	306.8	136.2	170.8
	Df	2	2	2
	p-value	0.00	0.00	0.00

Pairwise post-hoc comparisons using Wilcoxon Signed Ranked tests were performed to compare all possible pairs. Based on the post-hoc investigation there was a statistically significant difference in citation counts between all pairs (p < 0.001) except between Google Scholar and Scopus for CM physics 2003 (p = 0.119).

### Overlap and uniqueness of citing references

An examination was done of the citing references returned from 2003 for both oncology and CM physics to further examine the composition of these sets. (Scopus did not return sufficient material for 1993, and so only 2003 articles were examined in this portion of the study.) In particular, we wished to determine the amount of citing references unique to each index, and the amount of citing references occurring in two or all three resources. An automated matching algorithm was developed to identify the overlapping and unique citing references. For each article, the algorithm divided all of its citing references into seven groups:

1. Overlap of all three resources

2. Overlap between Web of Science and Google Scholar

3. Overlap between Web of Science and Scopus

4. Overlap between Google Scholar and Scopus

5. Unique references from Web of Science

6. Unique references from Scopus

7. Unique references from Google Scholar

**Figure 1:**
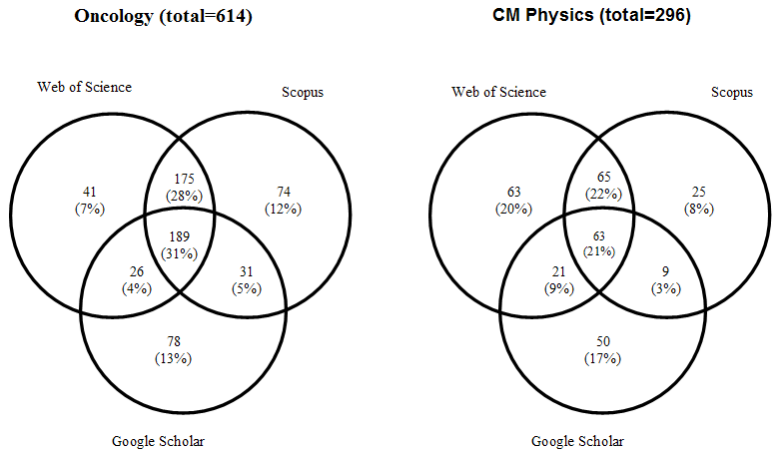
The distribution of the unique and overlapped citing articles as returned by the algorithm. Note: diagram is not to scale.

A sample of articles was selected to test the accuracy rate of the matching algorithm. The citing references for these articles were gathered, and the resulting set of 320 citing references were checked manually to determine if the algorithm had placed each citing reference in the correct category (of the seven listed above.) If the citing article was not placed in the correct category by the matching algorithm, it was marked as an error. The test demonstrated an accuracy rate of 98% for the matching algorithm. This was an acceptable accuracy rate, and so the matching algorithm was then used to categorize the citing references for all of the 2003 articles. Figure 1 shows the distribution of the unique and overlapping references as returned by the algorithm.

For oncology articles published in 2003, the greatest number of unique items was found in Google Scholar (78, 13%) followed by Scopus (74, 12%). A search of Web of Science revealed a much smaller number of unique citing articles (41, 7%). A core group of 189 (31%) citing articles was found in all three resources.

In the set of all citing material for the CM physics 2003 articles, the greatest amount of unique material was found in Web of Science (63, 21%), which also produced the highest citation counts. The second greatest concentration of unique material was in Google Scholar (50, 17%). Scopus returned 25 unique citing articles (9%). In contrast to the set of articles for oncology, only 63 (21%) were found in all three resources.

The large amount of unique material returned by Google Scholar led to further examination of the composition of that material. What percentage of that material would correspond to the traditional journal literature, and what percentage might reflect new forms of scholarly communication methods? From each 2003 set, 50 unique citing references from Google Scholar were examined to determine their origin. Citing references were classified as:

1. Journal: any journal, open access or not.

2. Archive: A subject-specific repository. Examples are arXiv (physics), Repec (economics) and ADS (astrophysics).

3. College or university sponsored: An institution-specific resource. May be a repository (such as the DSpace repository at MIT) or simply a departmental Web page.

4. Governmental: white papers and technical reports from .gov sites.

5. Non-governmental Organizations: White papers and technical reports from research institutes.

6. Commercial Entity: A paper published by a for-profit organization, such as a pharmaceutical company.

7. Other

The largest amount of unique material in Google Scholar came from journal literature for both oncology and CM physics. However in CM physics that percentage (38%) was much lower than for oncology (62%). In CM physics, the next largest contributing factor was material housed on archives (specifically arXiv). This accounted for 12 articles, or 25% of the unique material. In oncology, the next largest group of material came from colleges and universities (9 citing references, or 18%).

## Discussion

This study examined a defined set of articles from two subject disciplines: oncology and condensed matter physics. A search was done to uncover citing material in each of three products, Web of Science, Scopus and Google Scholar. For articles published in 1993, Web of Science returned the greatest number of citing articles in both CM physics and oncology. In oncology 1993, Scopus was next and Google Scholar produced the least number of citing articles. In CM physics 1993, Google Scholar was next and Scopus provided too few articles to study. Given the depth of Web of Science coverage in sciences (back to 1900), and Google Scholar's reliance on digital material (which in general dates back to the mid-1990's) this result is not surprising. For articles published in oncology in 2003, Scopus returned the highest number of citing references, followed by Web of Science and Google Scholar. In CM physics 2003, Web of Science returned the largest number of citing references, and the number returned by Scopus and Google Scholar was not statistically different. This result surprised the authors and contradicted their supposition that changes in scholarly publication, especially in CM physics, would be reflected in the citation counts from Google Scholar.

To look further at the sets of citing material returned, the unique and overlapping material found for both oncology and CM physics in 2003 were examined. In oncology, the largest set of unique material came from Google Scholar, but in CM physics Web of Science returned more unique material. In oncology, a larger percentage of citing material was common to all three resources (31%) than in physics, where only 21% of material was contained in all three resources. When unique citing references were found in a search of Scopus or Web of Science, it was either because of different journal title coverage, or sometimes because one index included articles from a particular publisher faster than the other. In Google Scholar the composition of the unique citing material was more varied, consisting of journals, e-prints, university, governmental and non-governmental material.

The overlap offered by Google Scholar was in some ways as interesting as the unique material. In oncology, 40% of the material it produced overlapped with either Scopus or Web of Science or both indices. The large amount of overlapping material gave some credence to the scholarly nature of the Google Scholar database, and the accuracy of its matching algorithm for detecting correct citing references. The unique material returned by Google Scholar sometimes consisted of journal literature not covered by the other indices (or not yet indexed) but also was comprised of a mix of material published on e-print archives, university, governmental and non-governmental organization web sites.

A possible bias may have been introduced by using JCR (a Thomson Science produced companion product to Web of Science) to select the journal titles for each discipline. One might wonder whether changing the study by selecting the journal titles from another source would have made an impact on the results reported in this study. However, the journals included in this study were indexed by Web of Science and Scopus, and appeared in Google Scholar; this argues against any possible bias created by using JCR.

During this investigation, it became obvious that Google Scholar changed rather dramatically after November 2005. When searches were rerun in Google Scholar in January 2006, some results were much larger. It can safely be assumed that Google Scholar citing result sets for the same articles studied here would now be different and probably larger. Similarly, some searches run in Scopus now give much higher citation counts. It would appear that Scopus has also undergone some improvements.

## Conclusion

This study analyzed the numbers of citing references for given sets of articles from journals in oncology and condensed matter physics for two publication years: 1993 and 2003, and further compared the citing references for fifty articles in two disciplines for 2003 only. This study did not identify any one of the three tools studied to be the answer to all citation tracking needs. Scopus showed strength in providing citing literature for more current (2003) oncology articles. However, Web of Science seemed to perform better for current CM physics, and was stronger for both subjects for articles published in 1993. Google Scholar returned a smaller number of citing references, but did provide a large set of unique citing material for 2003. Also, as a resource freely available to anyone with Internet connectivity, Google Scholar deserves consideration as an important adjunct to other research indices. This study however indicated that at this point Google Scholar alone might not replace other scholarly search tools. Scopus and Web of Science remain very important resources, but this study cannot claim one to be the clear winner for all subject matter. Rather, it indicates that the question of which tool is better, or at least which tool is better in terms of providing the most complete set of citing literature, may depend on the subject and publication date of a given article.

This study revealed that a researcher who needs to be comprehensive in a literature search has no simple solution. That is, none of these products covered the entire set of citing articles this study produced. In oncology, a researcher who consulted the index with the largest number of citing references (Scopus) would have found 76% of these citing references, and by adding a search of Google Scholar (which produced the most unique material) would find 94% of citing references. In CM physics a researcher who consulted only Web of Science would find 71% of citing references. By consulting Google Scholar in addition to Web of Science they would find 91%. A researcher using any two of three tools would find the majority of, but not all, citing material found in this study.

We note that Google Scholar and Scopus have both changed, perhaps dramatically since the time this sample was drawn. This would indicate that sampling should be repeated for more up-to-date comparisons, and to most fairly evaluate the utility of Google Scholar and Scopus. In addition, it is clear that Google Scholar provides unique citing material. The exact composition of this citing material should also be more thoroughly examined so that scholars will have a clear idea what is and is not included in Google Scholar searches.

## Competing interests

The author(s) declare that they have no competing interests.

## Authors' contributions

KB conceived the study, coordinated the project, participated in data collection and drafted most parts of the manuscript. NB participated in the data collection, performed the statistical analysis and interpretation of the data and helped with the draft of the manuscript. JG participated in the data collection, conducted literature review, and edited the draft of the manuscript. LW participated in the data collection, developed the Google Scholar citing reference extraction tool and the matching algorithm for grouping citing references. All authors read and approved the final manuscript.

**Table 6:** Composition of unique 2003 Google Scholar material

**Source of unique material**	**Oncology**	**CM Physics**
Journal	31 (62 %)	18 (37%)
Archive	3 (6%)	12 (25%)
College or University	9 (18%)	6 (13%)
Government	3 (6%)	4 (8%)
Non-Governmental Organization	2 (4%)	8 (17 %)
Commercial	0	0
Other	2 (4%)	0
Total	50	48

## References

[B1] Cheek J, Garnham B, Quan J (2006). What's in a number? issues in providing evidence of impact and quality of research(ers). Qual Health Res.

[B2] Seglen PO (1997). Why the impact factor of journals should not be used for evaluating research. Br Med J.

[B3] Walter G, Bloch S, Hunt G, Fisher K (2003). Counting on citations: A flawed way to measure quality. Med J Aust.

[B4] Kling R, McKim G (1999). Scholarly communication and the continuum of electronic publishing. J Am Soc Inf Sci.

[B5] Kling R, McKim G (2000). Not just a matter of time: Field differences and the shaping of electronic media in supporting scientific communication. J Am Soc Inf Sci.

[B6] Modlin IM, Adler G, Alexander K, Arnold RF, Brenner DA, Corazziari E, Floch MH, LaPorte RE, Peterson WL, Quigley EM, Shapiro MD, Spechler SJ, Spiller RC, Tytgat GN, Wiegers WF (2005). Information assimilation and distribution challenges and goals for real and virtual journals. J Clin Gastroenterol.

[B7] Brown CM (1999). Information seeking behavior of scientists in the electronic information age: Astronomers, chemists, mathematicians, and physicists. J Am Soc Inf Sci.

[B8] Brown C (2001). The e-volution of preprints in the scholarly communication of physicists and astronomers. J Am Soc Inf Sci Technol.

[B9] Deis LF, Goodman D (2005). Web of Science (2004 version) and Scopus. Charleston Advisor.

[B10] Felter LM (2005). Google Scholar, Scirus, and the scholarly search revolution. Searcher.

[B11] Giustani D, Barsky E (2005). A look at Google Scholar, PubMed, and Scirus: Comparisons and recommendations. JCHLA/JABSC.

[B12] Henderson J (2005). Google Scholar: A source for clinicians?. Can Med Assoc J.

[B13] Myhill M (2005). Google Scholar. Charleston Advisor.

[B14] Noruzi A (2005). Google Scholar: The new generation of citation indexes. Libri.

[B15] Notess GR (2005). Scholarly web searching: Google Scholar and Scirus. Online.

[B16] Roth DL (2005). The emergence of competitors to the science citation index and the Web of Science. Curr Sci.

[B17] Vine R (2006). Google Scholar. J Med Libr Assoc.

[B18] Almind TC, Ingwersen P (1997). Informetric analyses on the World Wide Web: Methodological approaches to webometrics. J Doc.

[B19] Borgman CL, Furner J (2002). Scholarly communication and bibliometrics. Annu Rev Inf Sci Technol.

[B20] Butler D (2004). Science searches shift up a gear as Google starts scholar engine. Nature.

[B21] Garfield E (1955). Citation indexes for science – new dimension in documentation through association of ideas. Science.

[B22] Garfield E (1979). Citation Indexing – Its Theory and Application in Science, Technology, and Humanities.

[B23] Garfield E (2005). The agony and the ecstasy – the history and meaning of the journal impact factor. International Congress on Peer Review and Biomedical Publication.

[B24] Kurtz MJ, Eichhorn G, Accomazzi A, Grant C, Demleitner M, Henneken E, Murray SS (2005). The effect of use and access on citations. Inf Process Manage.

[B25] Rahm E, Thor A (2005). Citation analysis of database publications. SIGMOD Rec.

[B26] Weale AR, Bailey M, Lear PA (2004). The level of non-citation of articles within a journal as a measure of quality: A comparison to the impact factor. BMC Med Res Methodol.

[B27] Zhao DZ (2005). Challenges of scholarly publications on the web to the evaluation of science – A comparison of author visibility on the web and in print journals. Inf Process Manage.

[B28] Vincent A, Ross D (2000). On evaluation of faculty research: Impact of citation analysis. J Appl Bus Res.

[B29] Bollen J, de Sompel HV, Smith JA, Luce R (2005). Toward alternative metrics of journal impact: A comparison of download and citation data. Inf Process Manage.

[B30] Holden G, Rosenberg G, Barker K (2005). Tracing thought through time and space: A selective review of bibliometrics in social work. Soc Work Health Care.

[B31] Bauer K, Bakkalbasi N (2005). An examination of citation counts in a new scholarly communication environment. D-Lib Mag.

